# From Immunohistochemistry to New Digital Ecosystems: A State-of-the-Art Biomarker Review for Precision Breast Cancer Medicine

**DOI:** 10.3390/cancers14143469

**Published:** 2022-07-17

**Authors:** Sean M. Hacking, Evgeny Yakirevich, Yihong Wang

**Affiliations:** Department of Pathology and Laboratory Medicine, Warren Alpert Medical School, Brown University, Rhode Island Hospital and Lifespan Medical Center, 593 Eddy Street, Providence, RI 02903, USA; hackingsean1@gmail.com (S.M.H.); eyakirevich@lifespan.org (E.Y.)

**Keywords:** digital ecosystems, breast cancer, Precision Medicine, biomarkers, state-of-the-art, review

## Abstract

**Simple Summary:**

In this state-of-the-art breast biomarker review, we have tried to imagine and illustrate future, emerging digital breast cancer ecosystems which allow for greater incorporation of traditional immunohistochemical and molecular biomarkers, WSI, and radiomic features.

**Abstract:**

Breast cancers represent complex ecosystem-like networks of malignant cells and their associated microenvironment. Estrogen receptor (ER), progesterone receptor (PR), and human epidermal growth factor receptor 2 (HER2) are biomarkers ubiquitous to clinical practice in evaluating prognosis and predicting response to therapy. Recent feats in breast cancer have led to a new digital era, and advanced clinical trials have resulted in a growing number of personalized therapies with corresponding biomarkers. In this state-of-the-art review, we included the latest 10-year updated recommendations for ER, PR, and HER2, along with the most salient information on tumor-infiltrating lymphocytes (TILs), Ki-67, PD-L1, and several prognostic/predictive biomarkers at genomic, transcriptomic, and proteomic levels recently developed for selection and optimization of breast cancer treatment. Looking forward, the multi-omic landscape of the tumor ecosystem could be integrated with computational findings from whole slide images and radiomics in predictive machine learning (ML) models. These are new digital ecosystems on the road to precision breast cancer medicine.

## 1. Introduction

Breast carcinoma is the most diagnosed malignancy in women. According to the Cancer Fact and Figure published in 2022 by the American Cancer Society, invasive breast cancer will be newly diagnosed in an estimated 287,850 women in the US in 2022, and result in an estimated 43,780 breast cancer deaths (43,250 in women, 530 in men) [1]. The overall female breast cancer incidence rates have increased by about 0.5% per year since the mid-2000s. Mortality has been declining since the 1990s because of earlier detection through screening, increased breast cancer awareness, and improved treatment. The important risk factors for breast cancer development are gender, age, lifetime exposure to estrogen, genetic inheritance, and environmental and lifestyle factors. Breast cancer is characterized by a high fat and protein diet combined with a lack of physical exercise. Obesity and its associated chronic inflammation were shown to increase the risk of obesity-driven breast cancer through leptin, adiponectin, estrogen, and several pro-inflammatory cytokines [2].

Environmental contaminants, such as organochlorine pesticides and certain plastics, have estrogenic effects on humans that may increase the risk of breast cancer [3]. Reproductive factors also affect breast cancer risk [4]. Early pregnancy (prior to 20 years of age) and prolonged breastfeeding are the major factors that decrease risk [5]. In contrast, delayed childbearing, fewer pregnancies, and reduced breastfeeding—combined with a lack of access to optimal health care- contribute to breast cancer incidence in low socioeconomic areas [1]. Alcohol consumption as low as 15–30 g/day has also been found to be associated with an estimated 30–50% increase in breast cancer risk [6].

Breast cancer represents complex ecosystems and is heterogeneous at radiologic, histologic, and molecular levels. Clinically, breast cancers are categorized into three major groups, and established from estrogen receptor (ER), progesterone receptor (PR), and human epidermal growth factor receptor 2 (HER2) expression. There are luminal (ER+ and HER2-), HER2-enriched (ER+ or −, and HER2+), and triple-negative (TN) breast cancers (ER- and HER2-). Luminal breast cancer is further divided into Luminal A (ER+, PR+/−, HER2-, Ki67 ≤ 14–20%)) and Luminal B (ER+/−, PR+/−, HER2+, or Ki67 > 14–20%). These breast cancer groups are distinct at genomic, transcriptomic, protein, and morphologic levels [7,8].

**Figure 1:**
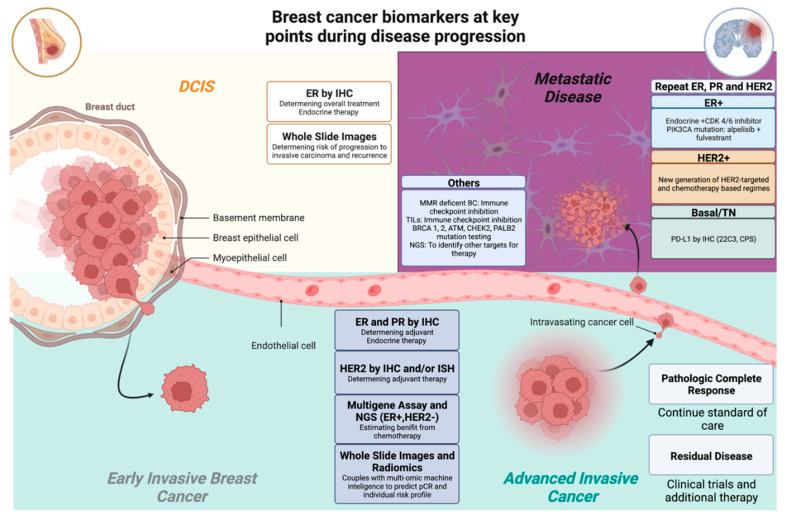
Breast cancer biomarkers at key points during disease progression. Adapted from the following references [10,11,12,13,14].

Breast cancer is evaluated by its histopathologic type, grade, size, lymph nodal status, and distant metastasis based on the traditional anatomic extent of disease (stage). The 8th edition of the American Joint Committee on Cancer (AJCC) Staging Manual integrates traditional anatomic staging with biological factors, including ER, PR, HER2, and multigene assay results, to create a Clinical Prognostic Stage Group [9,10] (Table 1). With an improving apprehension of the molecular landscape of breast cancer and the introduction of innovative targeted therapeutics, more biomarkers have been characterized to help clinical decision-making. In this state-of-the-art review, we summarize key 10-year updated recommendations from the American Society of Clinical Oncology (ASCO) and the College of American Pathologists (CAP), and highlight several emerging breast cancer biomarkers at genomic, transcriptomic, and proteomic levels, as well as whole slide imaging (WSI) and radiomics (Figure 1).

## 2. Hormone Receptors (HR)

**Figure 2:**
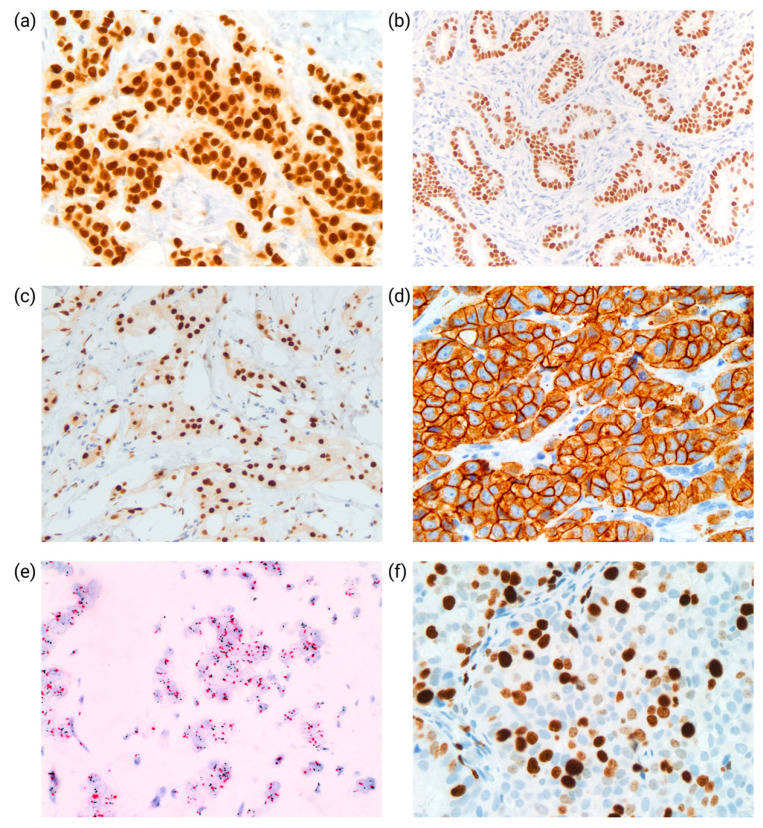
Biomarker analysis for subtyping of breast cancer. (**a**) ER expression by IHC; this is a positive ER with strong nuclear staining in >90%. (**b**) PR expression by IHC; this is a positive PR with moderate to strong nuclear staining in >90%. (**c**) AR expression by IHC; this is a positive AR with strong nuclear staining in >90%. (**d**) HER2 expression by IHC; this is a HER2 3+ positive with strong complete membranous immunoreactivity. (**e**) HER2 amplification by CISH; HER2-positive breast cancer identified by HER2 chromogenic in situ hybridization (CISH). The *HER2* gene copy number increase is detected using a *HER2*-specific probe (black signal), commonly co-hybridized to tumor cell nuclei using a second probe specific to the centromeric region of chromosome 17 (red signal). (**f**) Ki67 expression by IHC; nuclear stain highlights Ki-67 positive tumor cells. In this case, the Ki-67 proliferation index is estimated at 30%.

Most invasive breast cancers (75–80%) are hormone receptor (HR) positive, and estrogen receptor (ER)-positive tumors have been found to demonstrate improved survival following endocrine therapy [15]. The measurement of ER and progesterone receptors (PR) in breast cancers is performed to determine the potential clinical benefit of endocrine therapy. PR expression is more variable than ER expression, partially accounting for PR’s role in stratifying ER-positive cases into prognostic categories. ER and PR are evaluated on formalin-fixed, paraffin-embedded tissue sections via immunohistochemistry (IHC). Percentages are calculated by nuclear positivity for ER and PR reported according to a specified number or as a range. A *positive* result is equal to or greater than 1% of cell nuclei staining positivity (Figure 2a,b), a threshold that has been found to affirm the clinical survival benefit of endocrine therapy [11]. Recently, it has been acknowledged that growing evidence suggests that tumors with low ER expression have clinical survival and treatment response outcomes to chemotherapy like ER-negative cancers [15]. In the recent 2020 ASCO/CAP Guideline Update [16], a “low positive” category was added to the ER result interpretation. Thus, invasive breast cancer with 1–10% of cells staining for ER of any intensity is reported as “low positive”. This categorization enables those low-positive ER patients to be included in some triple-negative breast cancer clinical trials.

The androgen receptor (AR) is expressed in most breast cancers (70–85%) and is found primarily in ER-positive tumors [17,18] (Figure 2c). There is no guideline defining the AR threshold in breast cancer, and groups have used ≥1% or 10% tumor cells to define AR positivity [18,19,20]. The role of AR varies depending on the concurrent expression of ER and HER2. AR expression has been found to be associated with improved survival outcomes in ER-positive breast cancer [19]. Progress has been made in subdividing TNBC into distinct clinical and molecular subtypes based on the expression of AR along with comprehensive profiling of molecular features that could guide treatment decisions. In the Lehman classification, TNBC is subtyped into six groups. AR-positive TNBC is categorized as a luminal androgen receptor (LAR) subgroup, which exhibits a luminal-like gene expression pattern due to AR activation despite ER negativity and is responsive to PI3K/mTOR and anti-androgen inhibitors [21]. Burstein et al. [22] categorized TNBC into four distinct clinical subtypes: Luminal AR (LAR), Mesenchymal (MES), BL-immune suppressed (BLIS), and BL-immuno activated (BLIA). Newer classifications of TNBC based on molecular features and treatment response are promising and perhaps more pragmatic [23,24]. The GeparTrio trial revealed that tumors positive for AR had a lower likelihood of reaching pathological complete response (pCR) with neoadjuvant chemotherapy; however, tumors positive for AR had better clinical outcomes even without pCR [20]. AR is poised to be a promising therapeutic target for breast tumors, and numerous anti-androgen drugs are in the development pipeline; however, the role of AR as a predictive biomarker in clinical practice is uncertain [25].

## 3. Human Epidermal Growth Factor Receptor 2 (HER2)

HER2, or ERBB2, is a membrane receptor tyrosine kinase that promotes cell proliferation, development, and survival. Cancers with HER2 protein overexpression are usually due to gene amplification. HER2-positive is seen in around 20% of all breast tumors and may be seen with ER positivity and negativity. Clinically, HER2 is evaluated for protein overexpression detected by IHC (Figure 2d) or for gene amplification in situ hybridization, such as fluorescence in situ hybridization (FISH) or chromogenic in situ hybridization (CISH) (Figure 2e).

The ASCO/CAP issued the first recommendations for HER2 reporting in 2007. It was revised in 2013 and further updated in 2018 [26,27,28]. The most recent update (2018) changed the IHC 2+ equivocal definition from the 2013 version “circumferential membrane staining that is incomplete and/or weak/moderate, and within >10% of tumor cells,” to “weak to moderate complete membrane staining observed in >10% of tumor cells” (Table 2) [28]. In addition, the 2018 recommendations cover unusual staining patterns. An example is the micropapillary invasive carcinomas which sometimes exhibit a moderate to intense albeit incomplete membrane staining with HER2 gene amplification. This staining pattern in a micropapillary carcinoma is best classified as 2+ equivocal with reflux to in situ hybridization to determine the status of HER2 gene amplification [29].

In HER2 FISH or CISH testing, a chromosome enumeration probe (CEP17) is often included in determining the ratio of HER2 signals to copy numbers of chromosome 17. In the 2007 CAP/ASCO HER2 testing guideline, the HER2:CEP17 ratio was the only recommendation and mean HER2 signals/cell were utilized only when controls for the CEP17 probe were not present [26]. The 2007 guidelines also used a FISH “equivocal” when the HER2:CEP17 ratio was scored between 1.8 and 2.2. The updated 2013 guidelines later incorporated ratio and mean HER2 copy numbers for dual-probe ISH testing, with both a negative and positive category and an ISH equivocal category for cases with a ratio <2.0 and a mean HER2 signals/cell ranging between 4 and 6 [27].

The most recent 2018 update [28] includes five categories based on ratio and mean HER2 copy numbers (Table 2). Both the HER2 signal number and the HER2:CEP17 ratio are utilized to diagnose gene amplification. In most cases, both methodologies resulted in the same amplification status. Groups 1 and 5 are the most common. In categories 2–4, the status of ISH is determined in conjunction with the results of IHC, with a second reviewer reevaluating ISH if the IHC is 2+. The ISH result is interpreted with the concurrent IHC result to give a final positive and negative HER2 reading. This schematic allows IHC negative (0, 1+) and ISH categories 2–4 ISH to be reported as HER2 negative and HER2 positive IHC positive (3+). In the presence of equivocal IHC (2+), it is required that additional cell counting is performed to further determine ISH gene amplification status. If the ISH category 2–4 remains unchanged, HER2 status is reported as negative. This latest updated version is believed to cause less clinical confusion and ultimately results in a definitive positive or negative HER2 status. It is important to mention that clinical data pertaining to definitive response for HER2-targeted therapy in ISH categories 2–4 is limited.

Clinical trials evaluating novel therapeutics in the setting of metastatic breast cancer could utilize HER2, thus influencing future HER2 biomarker testing. Next-generation HER2-targeted drugs include trastuzumab-duocaramzine (SYD-985) and trastuzumab-deruxtecan (DS-8201), which have demonstrated promising results in HER2 “low” breast cancer [30]. Related clinical trials define “HER2 low” as negative for HER2 ISH with HER2 IHC scores of 1+ or 2+ [31,32]. This suggests that distinguishing between HER2 IHC scores of 0 and 1+ could become important for determining HER2 treatment eligibility.

Activation mutations in HER2 can also be targeted by drug therapies. However, activating HER2 mutations are only seen in 2–5% of breast tumors, most commonly in the setting IHC scores of 0–2+ and negative gene amplification by in situ hybridization. Such activating mutations are best identified by next-generation sequencing [33,34,35].

## 4. Ki67

Ki-67 is a nuclear protein and a biomarker for determining cellular proliferation. A MIB-1 monoclonal antibody is utilized to assess Ki-67 by IHC (Figure 2f). The percentage of Ki-67 positive tumor cells is often used to predict clinical outcomes for early-stage stage cancer and determine the need for adjuvant chemotherapy. In addition, it is also used for predicting chemotherapy activity and monitoring patients taking neoadjuvant endocrine or chemotherapy to determine the consideration of alternative therapies.

Ki-67 has also been shown to best distinguish luminal A and luminal B breast cancer molecular subtypes, with a cutoff of 14% [36,37]. In 2011, the 14% cutoff was adopted by a panel of experts during the St. Gallen International Breast Cancer Conference (2011) [38]. This same panel later voted to change the threshold to 20% or greater in 2013 [8].

Secondary to the confusion regarding defining low and high expression as well as a consensus cutoff in determining positivity and tremendous observer variability in the clinically relevant 10–20% range, the evaluation of Ki-67 as a biomarker has not received a recommendation from ASCO or the National Comprehensive Cancer Network (NCCN). Attempts to integrate Ki-67 into diagnostic workflows must be supported by data from clinical trials to support its potential for guiding therapeutic decisions.

Ki67 is useful in determining prognosis in ER+, HER2- breast cancers while identifying patient subgroups without the need for adjuvant chemotherapy. The phase III POETIC trial investigated postmenopausal women and found clinical outcomes associated with Ki67 following perioperative endocrine therapy in hormone-sensitive early breast cancer. Lower rates of recurrence were found in breast tumors harboring lower Ki-67 expression (<10%), suggesting that these patients may not need neoadjuvant endocrine therapy [39]. The ADAPT trial found that Ki67 proliferation indices could help identify patients who could be spared adjuvant chemotherapy following neoadjuvant endocrine therapy [40,41].

The monarch E clinical trial [40] investigated Ki-67 during a phase III trial of the adjuvant cyclin-dependent kinase inhibitor (CDKI) Abemaciclib. It established the benefit of adjuvant CDKI for HR+, HER2-, node-positive, early-stage breast cancers with higher recurrence risks for Ki-67 expression ≥20%. An updated monarch E trial reported that the Abemaciclib benefits were present more than two years following treatment, and the expression of Ki-67 ≥ 20% predicted prognosis, suggesting it may be utilized in conjunction with other clinical and pathologic findings to identify recurrence risk. However, while Ki-67 is prognostic, the study also found that the adjuvant abemaciclib + endocrine treatment did not result in benefits for patients identified to have high-risk clinical and pathological findings independent of the Ki-67 proliferative index [42].

Practically for pathology laboratories, Ki67 IHC has less value for determining treatments secondary to questionable preanalytical and analytical validities. Preanalytical variables, such as delay in fixation, can lead to decreased labeling [43]. The analytic validity for <5% to >30% is generally consistent; however, there is significant inter-observer/laboratory variability which is somewhere between >5% to <30% [44]. The *International Ki67 in Breast Cancer Working Group (IKWG)* has established and recommended adopting a standardized visual scoring method for clinical assessment [44,45]. It is worth mentioning that the Ki-67 biomarker has numerous counting methodologies. The IKWG now supports using average or global counting instead of “hot spot” methodologies, where only the highest proliferative areas are calculated. Hot spot methods have been found to be associated with more variability [44]. Ki-67 expression in the range of >5% to <30% by IHC supports the use of multigene expression assays such as Oncotype Dx, which is recommended by ASCO [16].

## 5. TILs and PD-L1

**Figure 3:**
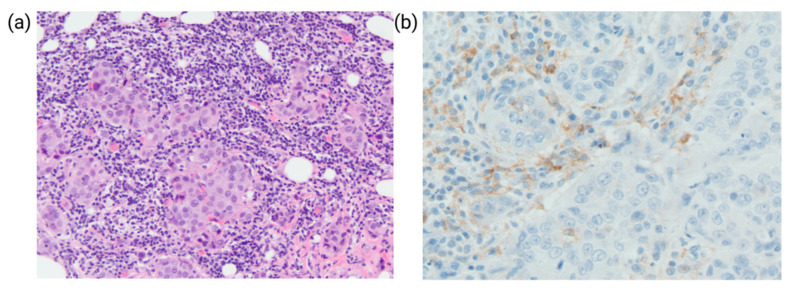
Tumor Infiltrating Lymphocytes and Programmed death ligand 1 (PD-L1) Expression in Breast cancer. (**a**) Invasive ductal carcinoma with dense lymphocytic infiltrate. (**b**) Positive PD-L1 expression in immune cells; tumor cells are negative.

The immune infiltrate associated with the tumors are referred to as tumor-infiltrating lymphocytes (TILs) (Figure 3a). TILs are mononuclear lymphocytes associated with tumor cells and the surrounding stroma and are thought to reflect the host immune response against the tumor. In recent years, with the increased interest in tumor immunotherapy and the intense investigation of the breast cancer immune microenvironment, TILs have emerged as a clinically important prognostic biomarker, as dense TILs have been shown to predict improved clinical outcomes and response rates to neoadjuvant treatment [46,47]. Recent studies also indicated that TILs predict response to immunotherapy and chemotherapy along with other targeted therapies [48,49].

To quantify TILs, it is recommended that one follow the international consensus scoring recommendation by *the Immuno-Oncology Biomarker Working Group* [50]. Reference images, digital slides, and guidelines for the interpretation of heterogeneous immune cells can be found at https://www.tilsinbreastcancer.org/pitfalls/ (accessed on 14 July 2022). In TNBC and HER2-positive cancers, stromal TILs (sTILs) quantification demonstrated reproducibility and predicted longer survival [46,47,51]; however, the significance of the TILs in ER-positive, HER2-negative tumors remains uncertain [51,52].

Programmed death ligand 1 (PD-L1) is one of breast cancer’s predictive biomarkers for immune checkpoint inhibitors (ICI) therapies. PD-L1 may be expressed on tumor cells and immunogenic tumor-infiltrating immune cells, including lymphocytes, macrophages, dendritic cells, and granulocytes (Figure 3b). Tumor cells upregulate the expression of PD-L1as a mechanism to evade the immune response and facilitate tumor development. PD-L1 binds to PD-1 on immune cells and promotes immune evasion and tumor progression, primarily by inhibiting cytotoxic T lymphocyte function [53]. The PD-1/PD-L1 interaction occurs between tumor and activated T-cells and represents the mechanistic pathway targeted by immunotherapeutic agents.

PD-L1 IHC testing is an emerging predictive biomarker to select patients with TNBC for immunotherapy. Currently, there are four Food and Drug Administration (FDA) approved companion diagnostics assays for PD-L1 in TNBC (SP142, 22C3, 28-8, and SP263) [54]. Those assays have different primary antibodies, detection systems, staining platforms, scoring criteria, and different cutoffs for PD-L1 positivity (>10% vs. >1%). The question is whether the drug should determine the assay used, or whether the result of the assay should determine the use of the drug? The two most studied PD-L1 assays, namely SP142 and 22C3, were companion diagnostics to select patients using the specific checkpoint inhibitors atezolizumab (monoclonal anti-PD-L1 antibody) and pembrolizumab (monoclonal anti-PD-1 antibody), respectively [55,56,57].

PD-L1 expression is evaluated using the combined positive score (CPS) for the 22C3 assay (Agilent Technologies, Santa Clara, CA). CPS is calculated as the number of PD-L1 stained cells, including both tumor cells and immune cells, which is divided by the total number of tumor cells multiplied by 100. Phase II KEYNOTE-086 studies showed significant value for immune cell infiltrates in relation to prediction prognostic outcomes in metastatic TNBC [55,56]. The phase III KEYNOTE-355 study contained 847 metastatic PNBC patients randomized and treated with pembrolizumab and chemotherapy vs. chemotherapy plus placebo and found pembrolizumab plus chemotherapy to be associated with better progression-free survival (PFS) for breast cancers with PD-L1 (22C3) CPS scores ≥10 in the immunotherapy arm [58].

The Impassion clinical trials used the SP142 assay (Roche Ventana, Tucson, AZ, USA). In this assay, tumor-infiltrating immune cells (IC) are reported as a proportion of total tumor area with PD-L1 staining in IC at any intensity. Positive PD-L1 expression is considered in tumors exhibiting ≥1% expression in IC [57]. In metastatic or locoregionally advanced TNBC phase III Impassion 130 studies, Schmid et al. demonstrated that additive atezolizumab in conjunction with nab-paclitaxel improved PFS in PD-L1 positive patients led to the FDA accelerated approval of atezolizumab in 2019 [57]. However, the subsequent IMpassion131 trial failed to meet the primary endpoint of progression-free survival (PFS) superiority in patients with PD-L1 positivity in frontline treatment, which led to the withdrawal of FDA approval in 2021 [59].

The posthoc analysis determined subgroup results for different PD-L1-based assays (including SP142, 22C3, and SP263), which suggested clinical superiority with SP142 immunopositivity. They also were able to demonstrate that in the presence of sTILs > 20% all patients had PD-L1 positivity, independent of which assay was utilized. These findings suggest that sTILs may drive immune response and could work to mitigate issues related to assay reproducibility during PD-L1 assessment [60,61].

For early-stage TNBCs, the GeparNuevo study utilized sTILs to stratify patients randomly into receiving durvalumab (a PD-L1 inhibitor) in conjunction with standard neoadjuvant chemotherapy and demonstrated that increased sTILs in bilateral study arms resulted in an increase in pCR status, but sTILs did not directly relate to durvalumab based response. Results from the IMpassion03 [62,63], KEYNOTE-522 [57], and I-SPY2 [64] (18) trials support that response rates to immune checkpoint inhibitors occurs independently to PD-L1 expression. Novel biomarkers with utility in predicting response for patients with TNBC in the setting of neoadjuvant chemotherapy and immune-checkpoint blockade could be beneficial. The use of TILs as a predictive biomarker and biomarker assay accuracy is under intense investigation.

## 6. Homologous Recombination

*BRCA1* and *BRCA2* are highly conserved pathways integral to double-strand DNA break repair and homologous recombination through the base excision repair pathway. Germline mutations in both the *BRCA1* and *BRCA2* genes are found in hereditary breast and ovarian cancers and account for around 7% of breast cancers with a predisposition toward TNBC status [65]. Somatic mutations in *BRCA1*/*BRCA2* have been shown to occur in 2.5% of all sporadic breast cancers [66]. *BRCA1* and *BRCA2* germline mutations are ubiquitous with deficient homologous recombination and respond favorably to PARP inhibitors [67,68]. However, emerging evidence suggests that a subset of *BRCA1*/*BRCA2* wild-type breast cancer patients can also have deficiencies in homologous recombination. Germline mutations in *PALB2*, *ATM*, and *CHEK2* [69] are also associated with breast cancer risks as high as those associated with BRCA1/2 mutations and are often referred to as *BRCA-like* breast cancer [67]. Lowry et al. [70] in a comparative modeling analysis demonstrated early annual MRI (age 30 to 35) with MRI and mammography starting at age 40 to decreased mortality associated with breast cancer by more than half (>50%) in women with germline *ATM, CHEK2*, and *PALB2* mutations.

Homologous recombination is a highly conserved process responsible for single-strand DNA break repair. Poly (ADP-ribose) polymerase (PARP) enzymes function and work to resolve stalled replication forks, and inhibiting PARP during the process of base excision could possibly repair BRCA-dependent homologous recombination [71]. Interestingly, the recruitment of nuclease MRE11 secondary to PARP1 activation has been hypothesized to influence DNA-end processing at DSBs, facilitate DNA repair-pathway choice, and channel DNA double-strand break (DSB) repair towards homologous recombination [72,73].

In a recent meta-analysis [74], PARP inhibitors were found to prolong progression-free and overall survival in patients with BRCA mutations and advanced breast cancers, with tolerable safety and overall improved quality of life. In breast cancers, two PARP inhibitors, olaparib and talazoparib, were recently approved in germline *BRCA* mutation *(gBRCAm*) carriers for the treatment of metastatic HER2-negative breast cancer based on both the OlympiAD [75] and EMBRACA [76] trials, respectively. The OlympiAD trial [75] was a large international randomized trial in *gBRCAm* carriers with HER2-negative breast cancer and a high risk of recurrence. The trial had to conclude early due to superiority in the olaparib arm, as a significant disease-free survival benefit was seen with lower rates of distant recurrence.

## 7. Mismatch Repair

Mismatch repair (MMR) represents the post-replicative process encoding both DNA homeostasis and genome stability [77], which functions to fix spontaneous base–base mispairing and small insertions–deletions (indels) that occur during DNA replication. Deficiency causes increased mutations and neoantigen loads [77]. MMR has a role in the cancer management in cancers of both colorectal and Mullerian systems and can identify patients and families at risk for Lynch syndrome [78].

Fusco et al. [79] demonstrated negativity for MMR by IHC (MLH1, MSH2, MSH6, and PMS2) to be 17% homogeneous and 12% heterogeneous in a cohort of 444 breast cancers. Luminal B-like breast cancers with MMR deficiency show worse clinical outcomes than MMR intact breast cancers, whereas negativity for ER-negative improved overall survival with MMR deficiency. Cheng et al. [80] demonstrated tumors lacking MMR showed worse clinical outcomes with ER positivity in the setting of tamoxifen treatment. It is worth mentioning that only three patients with MMR deficiency were detected by IHC in this study [80].

Hacking et al. [81] demonstrated MMR deficient breast cancer by Proteinarium [82] network analysis to show integrated clusters of histone hub genes, HER2-enriched, and TNBC status. It showed that MMR deficiency has poorer survival in patients with HER2-enriched breast cancers as compared with improved survival in TNBC. Future work evaluating MMR and its relationship to histone markers and cancer epigenetics may be of clinical benefit. Histone lysine methyltransferases (KMTs) are promising therapeutic targets; however, histone modification-based therapies and associated tumor responses are still preliminary [83]. The KMT nuclear receptor binding SET domain protein 2 (NSD2) is also associated with triple-negative breast cancer status and has been shown to regulate EGFR and ADAM9, a membrane-anchored protein in the ADAM (a disintegrin and metalloproteinase) family responsible for promoting HB-EGF [84].

Comparing MMR results from NGS with immunohistochemistry will be important for future studies to guide clinical practice workflows in breast cancer. Laboratory methods are transitioning from IHC to more advanced CGP. Immune checkpoint inhibition received approval for every MMR-deficient cancer subtype [85]. MMR mutational signatures have also been demonstrated to be associated with breast cancer brain metastasis [86], possibly supporting the testing of MMR and PD-L1 in this setting. Currently, the frequency of microsatellite instability-high (MSI-H) is reported to be extremely low (0–1.5%), although there is no standardized definition of MSI-H in breast cancer [87].

## 8. Multigene Assays

Advances in molecular diagnostic testing have demonstrated breast cancer to be a truly heterogeneous disease. Early transcriptome studies separated breast cancer into distinctive molecular subtypes based on the expression of distinct “intrinsic” genes, which differed in clinical outcomes [7]. Luminal A tumors were shown to have high expression of ER and other related genes, with a low proliferation index. Luminal B tumors were shown to express ER and have a high proliferation index with variable PR and HER2 expression. HER2-enriched breast cancer has been shown to express the HER2 oncoprotein and related genes with variable HR expression. Basal-like tumors express basal markers such as cytokeratin 5/6 and epidermal growth factor receptor (EGFR) and typically lack ER, PR, and HER2 expression (TN). The Basal/TN BC subtype is the most frequently found in BRCA1/2-associated breast cancer [88].

Gene expression signatures are also used in clinical decision-making in determining whether to administer chemotherapy in ER-positive, HER2-negative breast cancer. There are a variety of prognostic assays, such as Oncotype Dx, MammaPrint, Prosigna (PAM50), EndoPredict, Breast Cancer Index, and Genomic Grade Index. The 21 gene Oncotype Dx and 70 gene MammaPrint panels are the most widely used. Despite the many genes presented in these assays, recurrence risk scores are weighted and most heavily correlated with HR status and proliferation-related genes [89]. The Breast Cancer Index™ is now recognized by ASCO as the only genomic test to help determine the need for extended endocrine therapy after five years in early-stage HR+ breast cancer patients with node-negative or one to three positive nodes and no recurrence [12].

The most used gene expression assay in clinical practice is the Oncotype DX (Genomic Health, Redwood City, CA, USA). This 21-gene reverse transcriptase PCR assay analyzes 16 breast carcinoma-related and five reference genes to create a recurrence score (RS) ranging from 0 to 100. Initially, the Oncotype DX RS contained three groups: low-risk (<18), intermediate-risk (18–30), and high-risk (>30) [90]. It has a role as a prognostic biomarker in predicting the risk of recurrence at ten years and benefits from chemotherapy in ER-positive, HER2-negative breast cancer with 0–3 positive lymph nodes (N0-N1) [91]. For the low-risk group, endocrine therapy is sufficient, whereas for the high-risk group, benefits from chemotherapy surpass other potential side effects.

Additional studies for the intermediate-risk group [92,93] demonstrated that chemotherapy in women over 50 could be mitigated in the setting of an RS of 11–25 and women under 50 years of age with an RS of 11–16. Chemotherapy was found to be beneficial in women under the age of 50 with an RS of 21–25 or an RS of 16–20 with a coexisting high clinical risk factor such as high Nottingham grade and larger tumor size. At the present time, the Oncotype DX reporting is age-based and eliminates the previous intermediate category. The National Comprehensive Cancer Network (NCCN) recommends Oncotype DX as the preferred gene expression assay for predicting prognosis and chemotherapy benefit in N0/N1 postmenopausal, HR-positive, and HER2-negative breast cancer.

Recent pan-genomic studies evaluating DNA copy number, DNA methylation, exome sequencing, messenger RNA arrays, microRNA sequencing, and protein arrays have further categorized varied molecular profiles in different breast cancer subgroups [94]. The luminal A subtype is enriched in *GATA3*, *PIK3CA*, and *MAP3K1* mutations, while the basal-like are associated with *TP53* mutations. Other potentially targetable genomic alterations in BC, apart from *ERBB2 (HER2)*, include *PIK3CA*, *AKT1*, and *PTEN* mutations, and neurotrophic receptor tyrosine kinase (*NTRK*) fusions. In the metastatic setting, genomic alterations can also be associated with endocrine therapy resistance, including *ESR1* and *ERBB2* activating mutations, loss-of-function mutations in *NF1*, alterations in other MAPK pathway genes, and ER-related transcriptional regulators [95].

Comprehensive genomic profiling (CGP) utilizing next-generation sequencing (NGS) technology is used to detect therapeutically targetable genomic alterations in breast cancer for both primary and metastatic diseases [96]. Several CGP platforms, including FoundationOne, Caris Molecular Intelligence, and Tempus xT detect tumor mutational burden (TMB), microsatellite instability (MSI), and all other classes of genomic alterations. These included substitutions, insertions/deletions, gene fusions, and copy number alterations.

## 9. Liquid Breast Cancer Biopsy

The liquid biopsy, including circulating tumor cells (CTCs), circulating tumor DNA (ctDNA), cell-free RNA, tumor-educated platelets, as well as exosomes, is gradually becoming adopted in breast cancer clinical management [97,98]. CGP of ctDNA could optimize individual patient treatment and improve longitudinal screening [99]. The analysis of ctDNA and CTC plasma count following definitive cancer resection has been demonstrated to improve the timely detection of minimal residual disease, which could be used to guide adjuvant therapy and reduce rates of recurrence. The CTC plasma count has been shown to determine the stage and prognosis of breast cancer [98]. Cristofanilli et al. [99] demonstrated in 177 patients diagnosed with metastatic breast cancer that increased CTCs found in whole blood before treatment was associated with worse survival outcomes.

Kruspe et al. [100] demonstrated that probe activation by nucleases derived from CTCs could differentiate metastatic breast cancer with an area under the curve (AUC) of 0.851–0.903. The isolation of CTCs was performed using ScreenCell capture technology to collect malignant epithelial and stromal cells and separate them from other cells based on particle size. The current utility of such an assay is uncertain, especially considering that CTCs levels are usually low in lower-stage tumors.

The US FDA recently approved the Therascreen *PIK3CA* RGQ polymerase chain reaction assay as a companion diagnostic for detecting *PIK3CA* breast cancer mutations in both tissue and liquid biopsies, suggesting future applications for liquid breast cancer biopsy in clinical decision-making. Detection of *PIK3CA* mutations from ctDNA in locally recurrent unresectable or metastatic HR–positive and HER2-negative breast cancer can be treated with phosphatidylinositol 3-kinase inhibitor, alpelisib plus fulvestrant [101].

**Figure 4:**
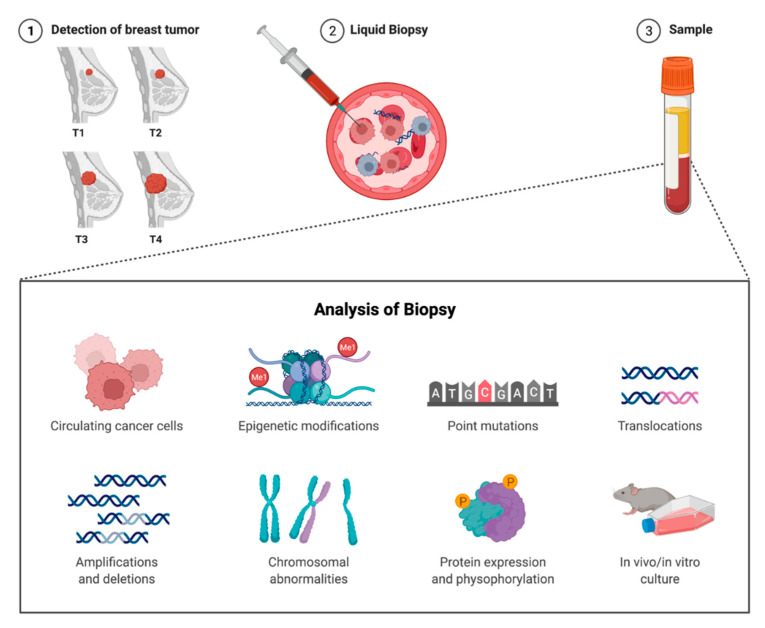
Overview of Liquid Breast Cancer Biopsy. (**1**) Breast cancer is detected either clinically or radiologically, (**2**) Liquid breast cancer biopsy is performed, (**3**) Samples can be analyzed for circulating cancer cells, epigenetic modifications, point mutations, translocations, amplifications and deletions, chromosomal abnormalities, protein expression and phosphorylation, and in-vivo/in vitro culture. Adapted from the following references [99,100,104].

Overall, the actual utility of liquid biopsies in breast cancer is yet to be defined. The concordance between liquid and tissue biopsies is an area of concern. Spoerke et al. [102] demonstrated in ER-positive metastatic breast cancer that the concordance between *ESR1* status in ctDNA and metastatic tissue was 47%, and 5% between ctDNA and primary tissue. *PIK3CA* mutations for ctDNA had a concordance of 86%, with tissue mutations determined by quantitative PCR. NGS platforms for liquid biopsy include the GeneStrat^®^ test (Boulder, CO, USA), PlasmaSELECT™-R 64 (Urbandale, IA, USA), InVisionSeq™ (Cambridge, UK), and Target Selector™ (San Diego, CA, USA), amongst others [103]. An overview of the liquid breast cancer biopsy’s theoretical implementation and testing applications is available in Figure 4.

## 10. Diagnostic Molecular Markers

Very few specific types of breast cancer are associated with single gene pathogenic alterations. Identification of the gene alterations is diagnostic for breast cancers such as tall cell carcinoma with reverse polarity and secretory carcinoma of the breast.

### 10.1. IDH Mutation in Tall Cell Carcinoma with Reverse Polarity

**Figure 5:**
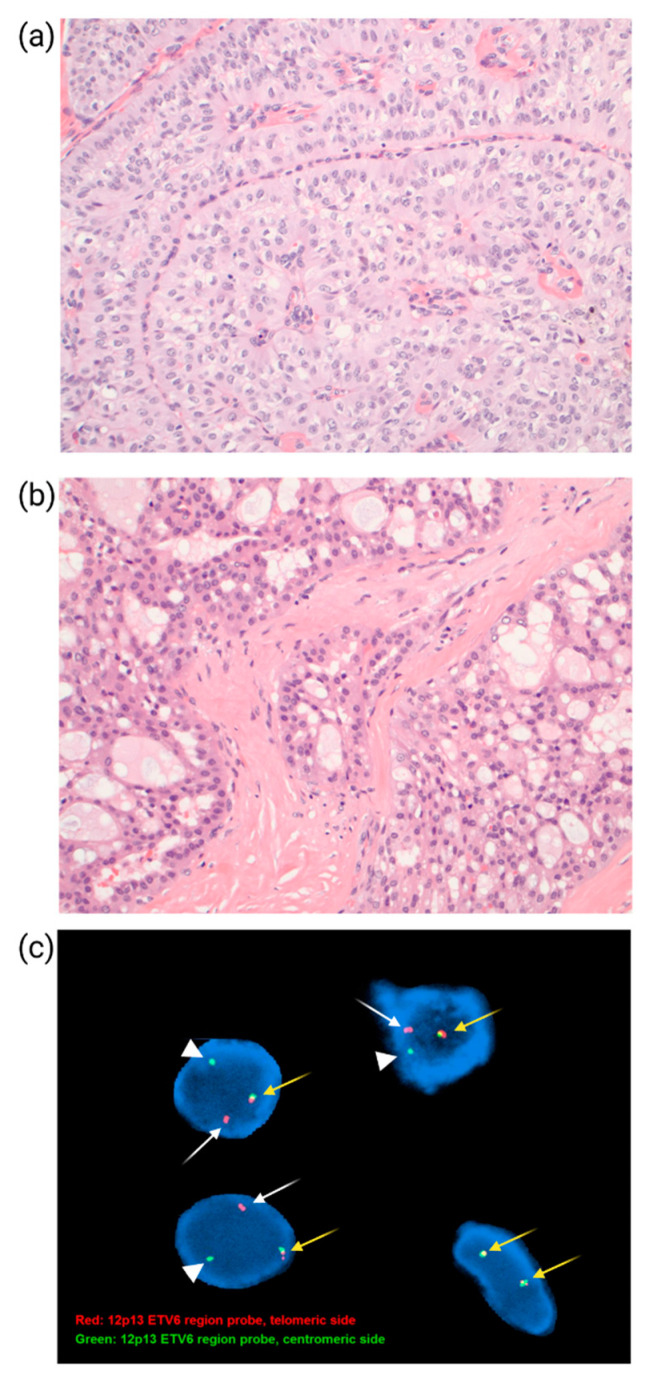
Breast carcinoma with specific features. (**a**) *Tall Cell Carcinoma with Reverse Polarity* with circumscribed nests of epithelial cells showing a solid papillary pattern and reverse polarity of nuclei located at the apical side of the tumor cells. (**b**) *Secretory carcinoma* with nests of tumor cells with ample cytoplasm and intracytoplasmic vacuoles. (**c**) *ETV6-NTRK3* gene fusion in the case of *secretory carcinoma*. Dual-color break-apart FISH studies of the tumor cells were performed with a 12p13 ETV6 telomeric probe (red) and a 12p13 ETV6 centromeric probe (green). Single red (arrow) and separate single green (arrowhead) signals demonstrate an ETV6 chromosomal rearrangement. An unsplit red and green probe signal demonstrates a non-rearranged wild-type ETV6 locus (yellow arrow).

*Tall Cell Carcinoma with Reverse Polarity* (TCCRP) is a rare type of breast tumor with solid papillary carcinoma feature and cytologically resembles the tall cell variant of papillary thyroid carcinoma [105,106]. Tumor cells are composed of circumscribed epithelial cell nests arranged in a dense fibrous stroma with a distinctive solid papillary histologic pattern. A prominent finding is apical nuclear polarity instead of the typical basal orientation of columnar epithelial nuclei; and this is referred to as reverse polarity (Figure 5a). TCCRP is most associated with triple-negative HR status and characteristic *IDH2* R172 hotspot mutations [106].

### 10.2. ETV6-NTRK3 Gene Fusions in Secretory Carcinoma

*Secretory carcinoma* is a rare histologic type of TNBC with a favorable prognosis. *Secretory carcinoma* is composed of epithelial cells with intracytoplasmic and extracellular secretions (Figure 5b) and has a unique, balanced translocation: t (12; 15) (p13; q25), with an *ETV6-NTRK3* gene fusion [107]. *ETV6* (TEL) encodes the *E26* transformation-specific transcription factor active in hematopoiesis and angiogenesis, whereas *NTRK3* encodes neurotrophic tyrosine receptor kinase 3, mostly expressed in the central nervous system. The Fusion protein is a chimeric tyrosine kinase with transforming activity. The same translocation occurs in adult acute myeloid leukemia along with other tumors such as mammary analogue secretory carcinoma of the salivary gland and pediatric mesenchymal tumors, including congenital fibrosarcoma and cellular mesoblastic nephroma, along with larotrectinib, a selective (tyrosine receptor kinase) TRK inhibitor, which is reported to be highly effective in treating solid tumors with *TRK* fusions [108]. However, for the secretory carcinoma of the breast, surgery alone is often sufficient to prevent recurrence and distant metastasis. Therefore, *ETV6-NTRK3* gene fusions are a diagnostic molecular marker for secretory breast carcinoma (Figure 5c).

## 11. Whole Slide Imaging, Radiomics and Multi-Omic Machine Learning

Advances in artificial intelligence (AI) coupled with growing digitization in the discipline of pathology are shouldering novel opportunities for profiling breast tumors with feature-engineered, computational approaches to whole slide images (WSIs) [13,109]. Recent reports in breast pathology have demonstrated the ability to quantify Ki67, detect mitosis, recognize lymph node metastasis, segment breast cancer tissue, assess TILs, and predict molecular profiles, treatment response, and prognosis from WSIs [13].

Beck et al. [110] first demonstrated survival predictors from WSIs to arise from tumor-associated stromata rather than the tumor epithelium. Stromal features associated with clinical outcomes were decreased variability between stromal matrix regions, contiguous regions of stromata contiguous lacking nuclei, and the increased complexity of stroma nuclear features.

WSI has also been utilized to spatially characterize patterns of breast cancer TILs. Fassler et al. [111] found that large aggregates of peritumoral and intratumoral TILs (forests) had improved clinical survival, while the lack of intratumoral TILs (deserts) correlated with tumor recurrence.

Most recently, WSIs have been identified to profile risk [112] and upgrade to invasive carcinoma [113] in patients with ductal carcinoma in situ (DCIS). Klimov et al. [112] developed a machine learning (ML) WSI pipeline in 344 patients treated with lumpectomy at Nottingham University Hospital, UK, with utility in predicting individual patient recurrence risk [112]. The recurrence classifier was able to predict a 10-year risk of recurrence in a training cohort (HR = 11.6) and an independent validation (HR = 6.39) cohort, indicating some degree of overfitting to the training data set. The most important computational feature was the quantification of hematoxylin staining, or blue color intensity, per pixel (or point) within the malignant ductal above a specified Otsu method [114] autogenerated threshold.

Hacking et al. [113] evaluated 44 mass-forming DCIS patients and found a high myxoid stromal ratio, low collagenous stromal percentage, and low proportionated stromal area to determine the risk of invasion following definitive surgery from core needle biopsy specimens. Future AI research is needed for mass-forming DCIS; however, the foregoing studies suggest that ML have significant utility. For invasive breast cancer, digital image analysis was found by Millar et al. [115] to show high stroma associated with a poor prognosis in TNBC and favorable prognostic outcomes in luminal A tumors [115].

Radiomics is a quantitative approach to medical diagnostic imaging aimed at improving existing data through advanced mathematical analysis [116]. Breast cancer radiomics is another emerging biomarker area for discussion [14]. Braman et al. [117] combined intratumoral and peritumoral radiomic features to produce an AUC of 0.74–0.78 for predicting pCR using a diagonal linear discriminant analysis (DLDA) classifier, part of the Naive Bayes classifier family, which assumes class distributions to be multivariate normal and to share a common covariance matrix [118]. The integration of HR status improved the AUC prediction for pCR by up to 0.83 in the HR-positive, HER2-negative group and 0.93 in the TN and HER2-positive groups by naive Bayes classifier. In HR-positive, HER2-negative tumors, a negative pCR status was associated with elevated peritumoral heterogeneity following initial contrast enhancement. On the other hand, TN and HER2-positive tumors which did not achieve pCR were found to have speckled enhancement patterns within the peritumoral regions. Radiomic features independently predicted pCR regardless of what type of ML was classifier used, supporting radiomics as a robust predictor of disease response.

Wang et al. [119] demonstrated that radiomic features are significantly associated with disease-free survival, and showed better predictive performance than traditional umor, Node, Metastasis (TNM) staging systems. They were also able to find distinct gene expression patterns, immunophenotype and immune cell compositions between radiomic features. They also found a link between radiomics and computational WSI features. Prior to this, the heterogeneity of background parenchymal enhancement, measured through quantitative texture features by *dynamic contrast-enhanced magnetic resonance imaging* (DCE-MRI), was found to strongly predict the TNBC subtype [120].

For invasive breast cancer and multi-omic ML, a deep learning-based genomics integrative framework was utilized by Malik et al. [121] to quantify drug response. The model predicted drug response based on a patient’s multi-omics features to common breast cancer drugs, including Dabrafenib, Gemcitabine, and a PI3K inhibitor (AS605240), amongst others.

Sammut et al. [122] collected clinical, WSI, genomic, and transcriptomic data from biopsy material of 168 breast cancer patients following neoadjuvant chemotherapy and correlated multi-omic features, thereby demonstrating that the multi-omic landscape of the tumor ecosystem could be integrated into predictive ML models. This included tumor mutational and copy number data, tumor proliferation, immune infiltration, and T cell aberrations, and were validated with other clinical data and WSIs in a separate cohort of 75 patients demonstrating an AUC of 0.87.

In a performance comparison performed by Asri et al. [123], different ML methods for breast cancer risk profiling and diagnosis were compared, showing support vector machine (SVM) to have the highest accuracy (97.13%) and the least associated error. In a systemic review by Li et al. [124] regarding the application of ML for the prediction of five-year survival rates in breast cancer, decision trees were shown to be most frequently deployed (61.3%), followed by deep learning (58.1%), support vector machines (51.6%), and ensemble learning (32.3%). An accuracy of 0.510–0.971, sensitivity of 0.037–1 and a specificity of 0.008–0.993 was seen with an AUC ranging from 0.500–0.972. Only 1 model performed external verification. For BC, transfer learning could introduce learned relationships to new ML classifiers, and ensemble learning could be used to combine the predictive power of numerous ML classifiers [125]. External verification will be important for truly determine the ability of an ML model to perform in a new clinical environment.

## 12. Conclusions

This review best suits the requirements of pathologists, oncologists, and researchers in the field who would want to benefit from an understanding of biomarkers indicative of breast cancer disease progression and categorization.

Breast biomarkers have been a rapidly evolving field in the past decade, highlighting the importance of reviewing the most recent 10-year updated recommendations. Testing for PIK3CA mutation, germline BRCA1/2, PALB2 pathologic variant, homologous recombination deficiency (HRD), PD-L1 expression, and TMB is some of the latest developments to guide breast cancer treatment. In some instances, there is no lack of controversy about the most accurate and appropriate testing for a biomarker, for example, the approval and withdrawal of the PD-L1 SP142 testing following the ongoing updated clinical trial results. Currently, ctDNA or circulating tumor cells are urgently needed to monitor patients’ responses to metastatic breast cancer. However, the data is still insufficient to support the clinical use of the testing. There is also a situation where treatment is available, for example, the TRK inhibitor. However, secretory carcinoma is rare, and the prognosis is good, so NTRK testing is unnecessary in breast cancer treatment decision-making.

Based on current guidelines, IHC functions as the workhorse of breast cancer immunophenotyping. Previous approaches for ML have been used to detect biomarker status from cellular morphology [126], which has also led to applications for virtual immunohistochemical staining [127]. Virtual IHC could forego the need for the wet lab in evaluating ER, PR, HER2, and Ki-67 in breast cancer, although this will require extensive validation. Developing robust metrics of quality assurance and quality control in both digital pathology and radiomics will be important in overcoming any limitations that could prevent a truly personalized treatment. The analysis of big data powered by next generation high-performance quantum computing could deliver new therapies and approaches based on effectively predicting individualized treatment efficacy and safety profiles for drugs and their targets.

**Figure 6:**
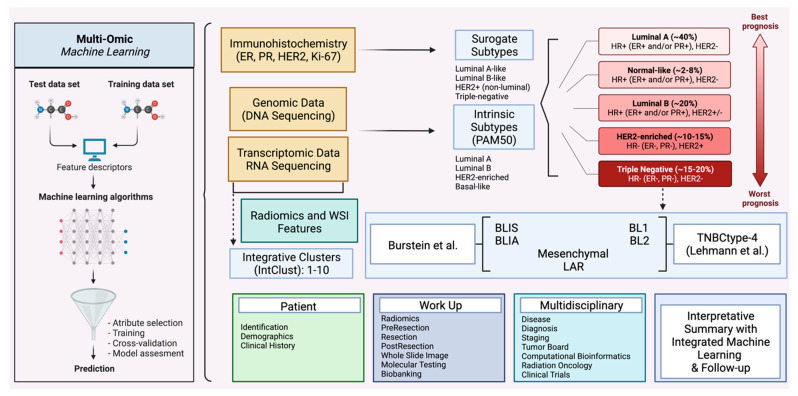
The precision breast cancer medicine digital ecosystem and diagnostic framework with integrated multi-omic machine learning. Immunohistochemistry is used as a surrogate marker, while genomic and transcriptomic data define intrinsic subtypes and integrative clusters of breast cancer with variable clinical significance. TNBC has the worst prognosis and contains subtypes such as luminal androgen receptor (AR; LAR), (ii) mesenchymal (MES), (iii) basal-like immunosuppressed (BLIS), and (iv) basal-like immune-activated (BLIA) as defined by Burstein et al. [22], and basal-like (BL1 and BL2) by Lehmann et al. [21]. The new digital ecosystem and diagnostic workflow rely on integrated ML, incorporating multi-omic features and reporting summary findings to clinicians in an understandable manner with follow-up. Adapted from the following references [21,22,128].

In this review, we have tried to imagine and illustrate future, emerging digital breast cancer ecosystems which allow for greater incorporation of molecular, WSI, and radiomic features. Such computational approaches could be applied with integrated ML and follow-up, allowing more precise information for individual breast cancer treatment planning. Pathologists, particularly breast pathologists, have long been critical in determining patient diagnoses and guiding management decisions by providing prognostic information. Pathologists can be integral to truly personalized patient breast cancer care through ongoing refinements in our fundamental understanding of the molecular mechanisms underlying the disease process and therapeutic response. Pathologists could also play a leading role in integrating the landscape of the tumor ecosystem with applications in multi-omic ML and reporting summary findings to clinicians and patients in an understandable manner with appropriate follow-up as necessary (Figure 6). This will lead to a more comprehensive characterization of breast tumors and promising strategies capable of optimizing patient clinical decision-making and providing better patient care in the era of precision breast cancer medicine.

## Figures and Tables

**Table 1:** Prognostic and Predictive Biomarkers in BC Staging in AJCC 8th.

Markers	Comments
Anatomic factors (TNM)	
Tumor (T)	Histologic type, grade, and size
Regional lymph nodes (N)	Nodal metastasis (including the number of involved nodes). The presence of nodal metastases is correlated with the probability of distant metastasis
Distant metastasis (M)	Metastasis beyond regional lymph nodes such as in organs like lung, bone, liver etc. Once distant metastases are present, a cure is unlikely, although long-term remissions and palliation can be achieved
Prognostic factors	
ER, PR, and HER2	All invasive tumors are tested for ER, PR, and HER2 status using IHC and ISH (for HER2)
Multigene Panels scores	Oncotype Dx recurrent score to identify patients with HR+, HER2- early stage breast cancer who can be spared the toxicity of chemotherapy

**Table 2:** HER2 Testing by Immunohistochemistry (IHC) and Dual-probe ISH Reporting.

Test Type	Score/ Group	Interpretation	Scoring Criteria (ASCO/CAP 2018)
**IHC**	Score 0	Negative	No staining observed, or membrane stating that is incomplete and is faint/barely perceptible and in ≤10% of tumor cells
	Score 1+	Negative	Incomplete membrane staining that is faint/barely perceptible and in >10% of tumor cells
	Score 2+	Equivocal	Weak to moderate complete membrane staining in >10% of tumor cells. Must order reflex test with ISH
	Score 3+	Positive	Circumferential membrane staining that is complete, intense and in >10% of tumor cells
**ISH**	Group 1	Positive	HER2/CEP17 ratio ≥2.0; average HER2 signals/cell ≥4.0
	Group 2	Negative *	HER2/CEP17 ratio ≥2.0; average HER2 signals/cell <4.0
	Group 3	Positive *	HER2/CEP17 ratio <2.0; average HER2 signals/cell ≥6.0
	Group 4	Negative *	HER2/CEP17 ratio <2.0; average HER2 signals/cell ≥4.0 and <6.0
	Group 5	Negative	HER2/CEP17 ratio <2.0; average HER2 signals/cell <4.0

* The final interpretation needs to take consideration with the HER2 IHC result.
